# Sounding out maerl sediment thickness: an integrated data approach

**DOI:** 10.1038/s41598-024-55324-x

**Published:** 2024-03-03

**Authors:** Jack Sheehy, Richard Bates, Michael Bell, Jo Porter

**Affiliations:** 1 International Centre for Island Technology, Heriot-Watt University, Orkney Campus, Robert Rendall Building, Franklin Road, Stromness, Orkney KW16 3AW Scotland; 2 School of Earth and Environmental Sciences, University of St Andrews, Bute Building, Queen’s Terrace, St Andrews, KY16 9TS Scotland

**Keywords:** Blue carbon, Eco-social-economics, Policy, Seagrass, Saltmarsh, Mangroves, Climate change, Imaging, Climate change, Ocean sciences, Climate-change ecology

## Abstract

Maerl beds are listed as a priority marine feature in Scotland. They are noted for creating suitable benthic habitat for diverse communities of fauna and flora and in supporting a wide array of ecosystem services. Within the context of climate change, they are also recognised as a potential blue carbon habitat through sequestration of carbon in living biomass and underlying sediment. There are, however, significant data gaps on the potential of maerl carbon sequestration which impede inclusion in blue carbon policy frameworks. Key data gaps include sediment thickness, from which carbon content is extrapolated. There are additional logistical and financial barriers associated with quantification methods that aim to address these data gaps. This study investigates the use of sub-bottom profiling (SBP) to lessen financial and logistical constraints of maerl bed sediment thickness estimation and regional blue carbon quantification. SBP data were cross validated with cores, other SBP data on blue carbon sediments, and analysed with expert input. Combining SBP data with estimates of habitat health (as % cover) from drop-down video (DDV) data, and regional abiotic data, this study also elucidates links between abiotic and biotic factors in determining maerl habitat health and maerl sediment thickness through pathway analysis in structural equation modelling (SEM). SBP data were proved to be sufficiently robust for identification of maerl sediments when corroborated with core data. SBP and DDV data of maerl bed habitats in Orkney exhibited some positive correlations of sediment thickness with maerl % cover. The average maerl bed sediment thickness was 1.08 m across all ranges of habitat health. SEM analysis revealed maerl bed habitat health was strongly determined by abiotic factors. Maerl habitat health had a separate positive effect on maerl bed sediment thickness.

## Introduction

Maerl, and rhodolith beds, are habitat forming species which alter the benthic substrate. They promote biodiversity and ecosystem function by providing suitable habitat for a wide range of fauna and flora^1^. Maerl sensitivity to disturbance and importance to biodiversity have highlighted the need for environmental protection^2,3^ and they are recognised as a priority marine feature (PMF) in Scotland^4^. Maerl also has the potential to sequester carbon and in Scotland are considered a ‘blue carbon’ species^5,6^. Due to data gaps on the scale and permanence of maerl carbon stocks^7^, however, maerl is not currently recognised in international frameworks as a blue carbon habitat; these are restricted to seagrass, saltmarsh, and mangroves^8,9^. There is also uncertainty on the export of autochthonous carbon, capture of allochthonous carbon, and the identification of these carbon fluxes in blue carbon habitats and wider marine ecosystem systems and sediments^10^. Research that clarifies uncertainty surrounding the carbon sequestration potential of maerl beds may support their integration into blue carbon frameworks, ecological conservation, and climate change mitigation.

In blue carbon literature the main determining factors of carbon sequestration potential are determined primarily by sediment characteristics, then abiotic factors, and finally by biotic inputs^11^. Identifying the relative importance of these factors, and their interactive effects, are essential to medium to large scale quantification of carbon stocks^12,13^. Small, medium, and large scale are defined as 0.05–0.1 km^2^, 1 km^2^, and > 1000 km^2^ respectively^12^. Maerl presence is principally correlated with moderate water flow and wave shelter^14,15^, but is generally insensitive to substrate type and found on fine mud, coarse gravel, and pebbles^16^.

Abiotic factors are also deterministic in maerl morphology and associated biodiversity^17,18^. Healthy maerl beds can support over 150 macroalgal species in addition to 500 faunal species^14,16^, may act as ‘marine seedbanks’ for regional biodiversity^19^, and support wider community resilience following disturbance^20^. Associated epiflora are important in maintaining maerl stability and persistence; both the maerl species and associated stabilising algae can be considered keystone species in maerl beds^16^.

Productivity, respiration, and calcification processes of the maerl itself, and associated fauna and flora, all determine whether the habitat is a carbon source or sink^11,21^. As with other blue carbon habitats, there is a large export of autochthonous carbon and import/burial of allochthonous carbon^22^. Habitat health has not, however, been investigated for its importance in determining maerl bed sediment thickness. Maerl habitat health may be determined by the general condition of the habitat (in relation to pollution, competition, and/or environmental stressors), and by local environmental suitability for the maerl habitat^23^. Benthic habitat health may be characterised by associated biodiversity^24^ but is more commonly referenced as surface biomass or % cover^25–27^ and/or abundance^28^; this study uses the latter definition and maerl % cover as a proxy for habitat health/condition.

The interaction of abiotic and biotic factors on carbon sequestration potential integrates ecological health with ecosystem service evaluation^29,30^, in addition to biodiversity values into carbon accounting processes^31^. This aligns with the wider policy context of eco-social economics; linking ecologic, economic, and societal values for climate change mitigation and adaptation^7^. Considering these values together, and the need for bottom-up societal engagement for blue carbon conservation, there is a subsequent requirement for cost-effective methods of quantification to resolve financial and logistical constraints of blue carbon accounting^12,32^.

There are a number of methods used in blue carbon science to identify and quantify marine carbon^33–36^. Different methods are subject to unique financial and logistical constraints^12^; this can either result in the best method being dependant on the end research goal, or that operational constraints dictate the scope of the research goals^7,16^. Subsequently, this can skew the focus of blue carbon research and impede marine conservation efforts^7^. This may be resolved through tier level assessment^33^, with guidelines that recommend average rates of autochthonous and allochthonous inputs^10^, and adaptive valuation^7^. There is still, however, a distinct need for cost-effective, easily applicable, quantification methods at scale to support blue carbon research. When considering regional surveys, significant data gaps on maerl sediment thickness, mostly limited to 0.6 m, impede robust estimates^5,37^.

This study investigates the feasibility of sub-bottom profiling (SBP) in determining maerl bed sediment thickness. This furthers limited research of acoustic sounding techniques in blue carbon science^36,38–42^; depth sounders have also been used to map areal extents of maerl habitats^16,43,44^. Acoustic sounding methods have the potential to provide small to medium scale data across the entirety of a seagrass habitat^12^ whilst most blue carbon data is focused on coring which provides one location specific data point^34^. When linked to cores, SBP data can extrapolate sediment layers and thickness across the entirety of the habitat. If also combined with estimates of habitat health, either as % cover^25–27^, biodiversity^3^, or structural complexity^23^, this provides a means to investigate the links between maerl habitat health and sediment thickness^13,45^; this study uses maerl % cover as a proxy for habitat health. This integrated approach supports cost-effective, easily applicable, small to medium scale quantification of blue carbon resources and by extension community level societal engagement to protect marine habitats^7^. When combined with regional abiotic data it supports analysis of the determining abiotic and biotic factors of maerl sediment stocks, and subsequently supports future research needs of machine learning approaches and large-scale assessment of regional blue carbon stocks^7,12,13,45^. This aligns with existing programmes to match abiotic and biotic factors to maerl presence and associated biodiversity^46^.

Maerl is commonly distributed around the UK, however, identification of specific maerl species requires genetic testing^47^. There are, therefore, limited estimates for total population coverage and extent. The two most dominant species of maerl in the UK are *Phymatolithon calcareum* and *Lithothamnion corallioides*; *corallioides*; though these classifications are under scrutiny in current genotypic research^48^. Due to their similarity and difficulty in individual identification, *P. calcareum*, *L. corallioides*, and *Lithothamnion glaciale* are often grouped together in literature^47^. Due to distributional differences of maerl species in the UK, with *L. corallioides* typically constrained to the south of the UK^14^, this paper only references *P. calcareum* and *L. glaciale* together collectively as maerl. In Scotland, maerl beds are common on the west coast in addition to Orkney and Shetland and account for approximately 30% of the maerl beds in north-west Europe^15^. In Orkney, there is an estimated 36.45 km^2^ of maerl bed but this estimate is subject to large uncertainty. Limited data also constrains estimates of maerl carbon content in underlying sediment to be generally constrained to a thickness of 0.25 m^5^.

The overall aim of this study is to determine the feasibility and robustness of SBP data in characterising maerl bed sediment thickness and to investigate the interactions of abiotic and biotic factors on maerl bed sediment thickness. This investigation has multiple objectives. These objectives include presenting the feasibility of SBP data to detail maerl bed sediment thickness and to demonstrate the use of SBP data for mapping maerl bed sediment thickness. This study then integrates SBP data to determine the relative influences and interactions of environmental variables and maerl bed habitat health on maerl sediment thickness. Finally, this study aims to highlight key areas of future research for quantification of maerl bed sediments within the context of blue carbon science and accreditation processes.

## Results

### Maerl % cover and sediment thickness

At Shapinsay the mean maerl Shallow Sediment Thickness (hereafter referred to as the SST) was 0.68 m (n = 44, SD = 0.07) with a maximum recorded thickness of 0.83 m at 80% maerl cover. The mean Total Sediment Thickness (hereafter referred to as the TST) for Shapinsay was 1.08 m (n = 44, SD = 0.11) with a maximum recorded thickness of 1.31 m at 60% maerl cover. Mean Wyre maerl SST was 0.79 m (n = 102, SD = 0.22) with a maximum recorded thickness of 1.35 m at 40% maerl cover. The mean TST for Wyre was 0.87 m (n = 102, SD = 0.21) with a maximum recorded thickness of 1.32 m at 40% cover.

**Figure 1:**
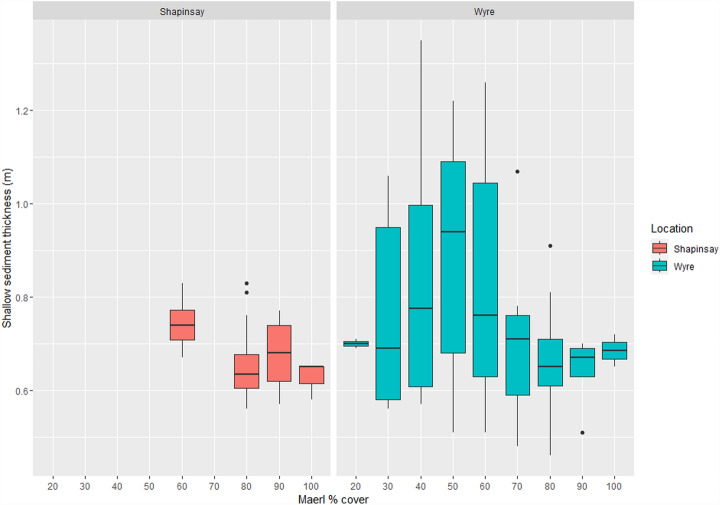
Shapinsay and Wyre maerl % cover against shallow sediment thickness (SST) boxplots.

**Figure 2:**
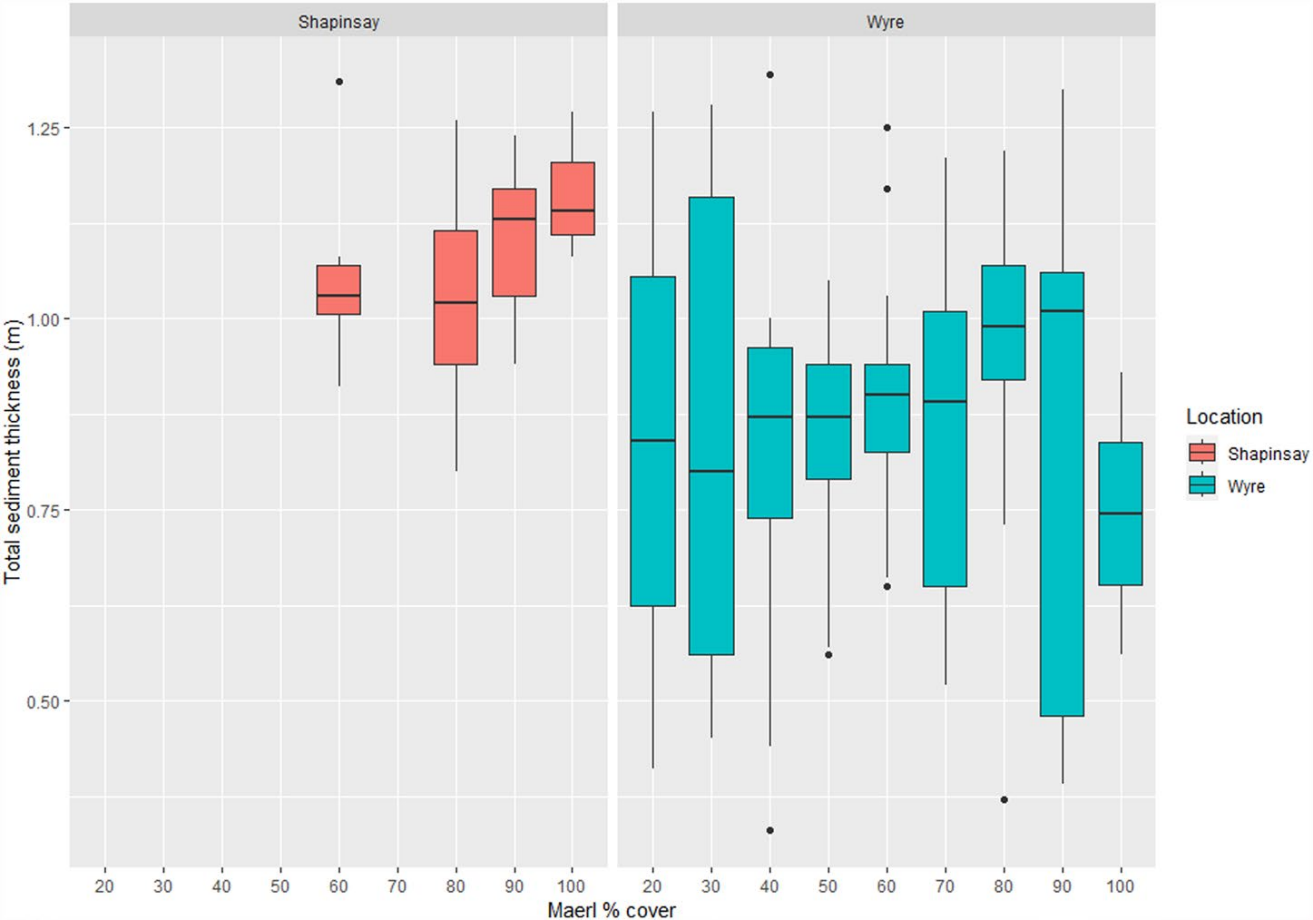
Shapinsay and Wyre maerl % cover against total sediment thickness (TST) boxplots.

There are limited data for Shapinsay maerl % cover, as seen in Figs. 1 and 2, with values only recorded from 60 to 100% cover, and no values for 70% cover. The Wyre subset of data exhibits a nearly full range of maerl % cover values from 20 to 100% in Figs. 1 and 2.

### Structural equation modelling (SEM)

**Figure 3:**
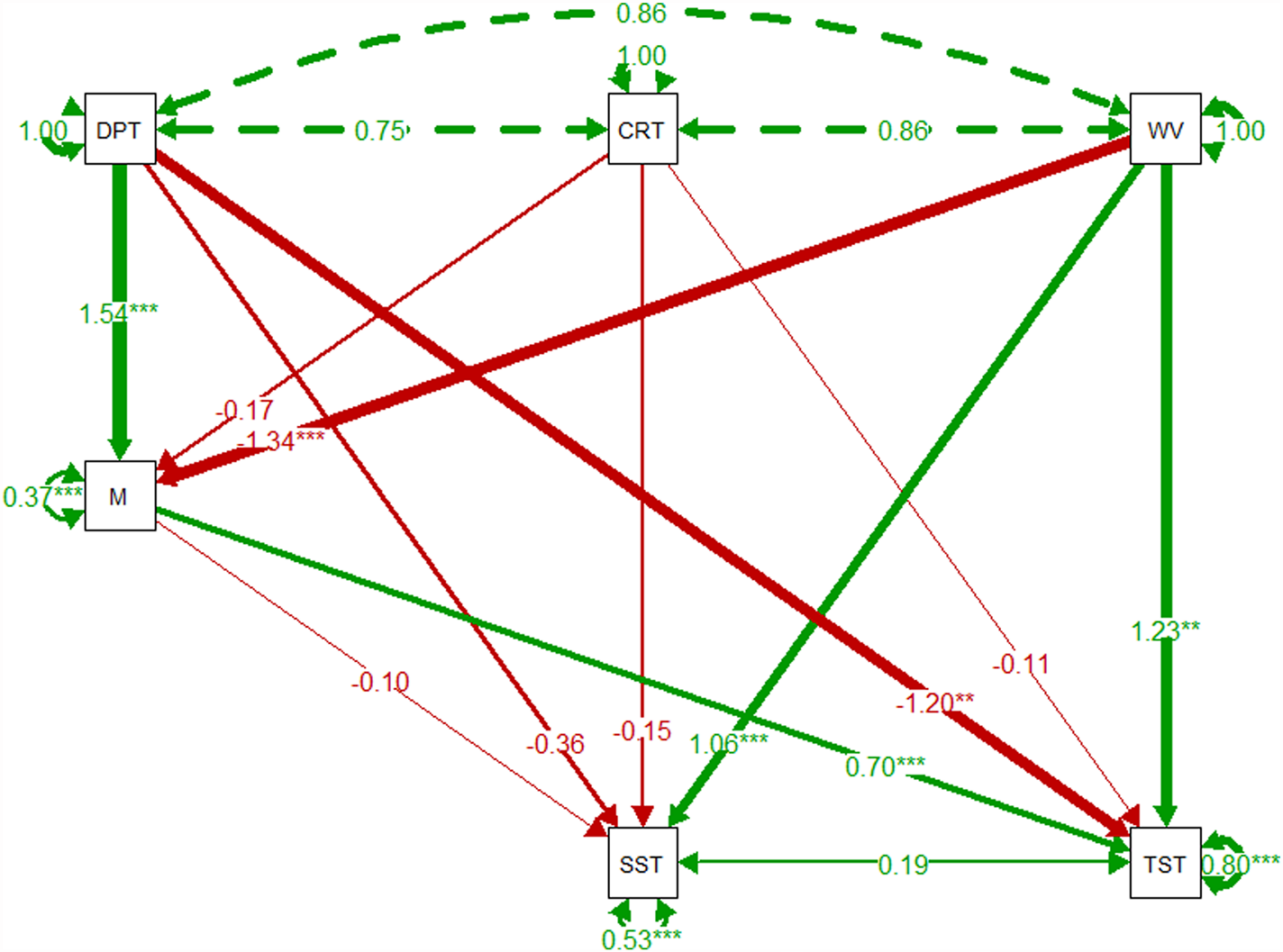
Shapinsay pathway SEMs of environmental variables, maerl % cover, shallow sediment thickness (SST), and total sediment thickness (TST). Nodes use abbreviations: *DPT* depth, *CRT* current, *WV* wave, *M* maerl % cover, *SST* shallow sediment thickness, and *TST* total sediment thickness. Green and red arrows denote positive and negative relationships respectively, dashed arrows represent covariance between exogenous variables. Arrow width denotes the coefficient strength. Circular arrows from each variable represent the residual variance. Coefficient estimate strength is detailed by the number along the arrow, with significance denoted by *p < 0.05, **p < 0.01, and ***p < 0.001.

The Shapinsay SEM shows strong covariance between all abiotic variables; depth, current speed, and wave exposure in Fig. 3. Shapinsay maerl % cover had a significant positive relationship with depth (1.54, *p* < 0.001). Wave exposure had a significant negative relationship with maerl % cover (− 1.34, *p* < 0.001). Only wave exposure had a significant relationship with the SST, with a positive correlation observed (1.06, *p* < 0.001). Depth was significantly negatively correlated with TST (− 1.20**). Wave exposure (1.23, *p* < 0.001) had a significant positive relationship with the TST. The most significant relationship with the TST, however, was observed in a positive correlation with maerl % cover (0.70, *p* < 0.001). The SEM of the Shapinsay subset of data is detailed in Fig. 3.

**Figure 4:**
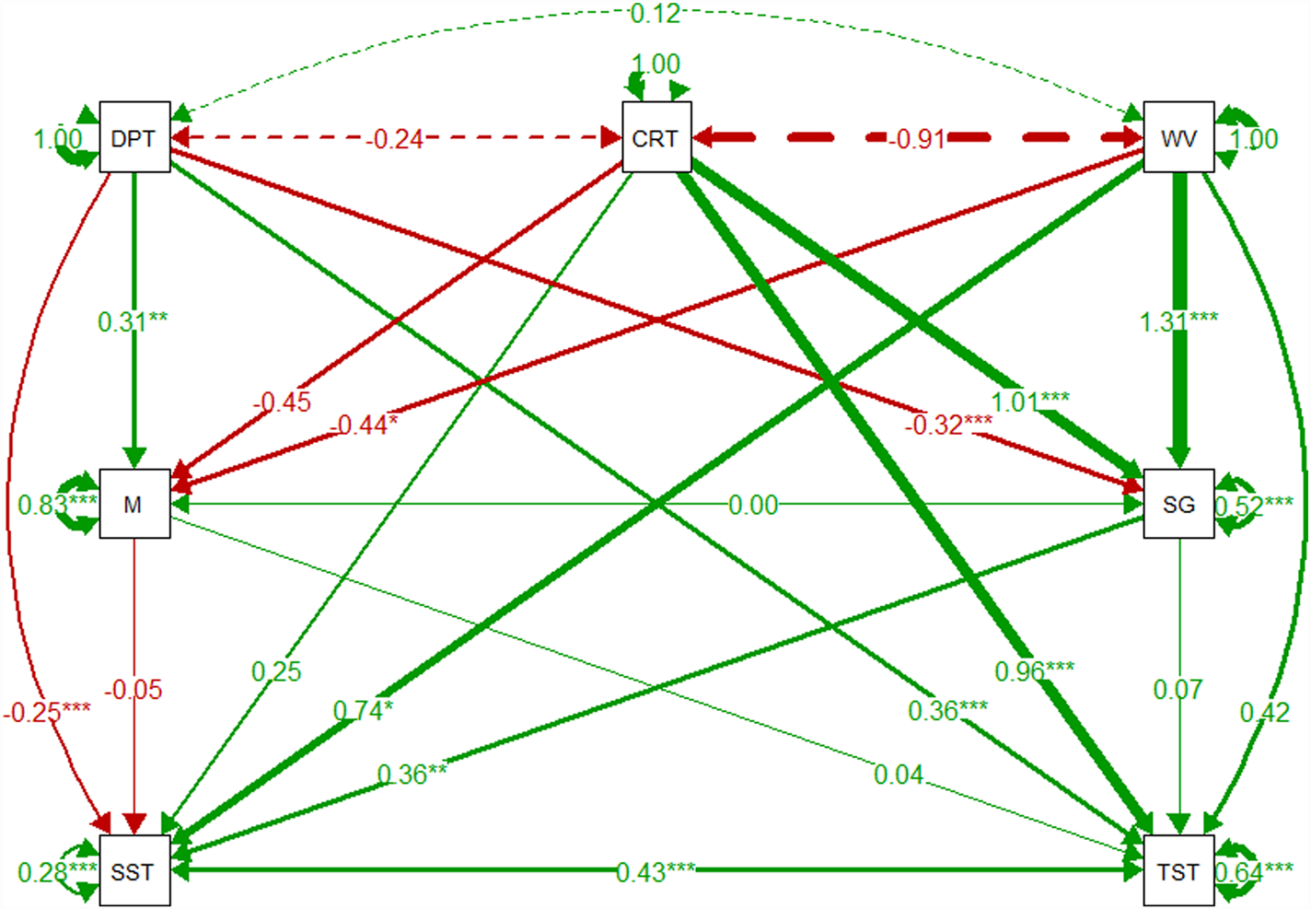
Wyre pathway SEMs of environmental variables, maerl % cover, seagrass % cover, shallow sediment thickness (SST), and total sediment thickness (TST). Nodes use abbreviations: *DPT* depth, *CRT* current, *WV* wave, *M* maerl % cover, *SG* seagrass % cover, *SST* shallow sediment thickness, and *TST* total sediment thickness. Green and red arrows denote positive and negative relationships respectively, dashed arrows represent covariance between exogenous variables. Arrow width denotes the coefficient strength. Circular arrows from each variable represent the residual variance. Coefficient estimate strength is detailed by the number along the arrow, with significance denoted by *p < 0.05, **p < 0.01, and ***p < 0.001.

The Wyre SEM shows little covariance between abiotic variables, only current speed and wave exposure show a strong negative correlation in Fig. 4. Wyre maerl % cover has a significant positive relationship with depth (0.31, *p* < 0.01) and a significant negative relationship with wave exposure (− 0.44, *p* < 0.05). Wyre seagrass % cover had a significant negative correlation with depth (− 0.32, *p* < 0.001) and was found to have significant positive correlations with current speed (1.01, *p* < 0.001) and wave exposure (1.31, *p* < 0.001). The SST had a significant negative correlation with depth (− 0.25, *p* < 0.001). Wave exposure (0.74, *p* < 0.05) had a significant positive correlation with the SST. Seagrass % cover (0.36, *p* < 0.01) had a positive significant relationship with the SST. Depth (0.36, *p* < 0.001) and current speed (0.96, *p* < 0.001) were found to have significant positive correlations with the TST. The SEM of the Wyre subset of data is detailed in Fig. 4.

## Discussion

### Sub-bottom profiling for blue carbon quantification

When aligned to local core data, this study finds SBP data to be an acceptably robust method to quantify maerl sediment thickness. Variability in maerl sediment thickness was evident with changes in % cover suggesting SBP data was accurate in capturing sediment layers. Sediment layer patterns identified from core data^49^ could also be aligned with SBP data with specific settings. Maerl bed sediment thickness is generally constrained with various values reported in the literature; 0.25 m^5^, 0.54–0.60 m^37,50^, 0.71–0.86 m^22^, and 1.175 m in Wyre^5^. As such, this study presents data that supports the use of SBP data for sediment thickness quantification, highlights maerl sediment layers, details maerl sediment thickness up to 1.35 m, and details mean sediment thickness of 1.08 m for typical maerl habitats on mixed substrate. Perhaps most importantly, this study details the use of SBP data to quantify maerl sediment thickness across small to medium scales and links sediment thickness to measures of habitat health and associated ecological function.

Using the results of SBP data is a relatively novel approach to quantify blue carbon sediment thickness, and has mostly been constrained as a quantification method for seagrass sediment^38–40,51^; there are no known applications of SBP for quantification of maerl sediment thickness. As such, it is unclear how robust SBP data may be in a wider context or be readily applied across biogeographic boundaries.

### Determining abiotic and biotic influences on maerl sediment thickness

The Shapinsay maerl site exhibits characteristics, mixed sediment substrate^52^, environmental variables, and associated fauna and flora typical of maerl habitats^53,54^. This site is therefore believed to be a good representation of other typical maerl habitats in Orkney and Scotland.

This subset of data, however, is slightly constrained in its range when accounting for spatial autocorrelation (n = 44). Maerl % cover values are also limited, and only cover 60–100%, excluding 70% cover; this may obscure robust analysis and extrapolation of these data to wider research. It is unclear whether the limited range of maerl % cover highlights a feature of maerl habitats with hard boundaries, rather than a gradual decline in maerl % cover, or is a result of sampling limitations.

The SEMs show the significance of abiotic factors, depth and wave exposure, on maerl % cover which align with known deterministic abiotic factors of maerl habitats^54^. Abiotic factors were variably significant in determining the SST and the TST. The SST had limited significant correlations, with depth having no observed relationship with the SST. This is likely due to depth, and linked availability of light, having a greater impact in structuring the live maerl sediment layers up to 0.35 m, which unfortunately were not sufficiently robust to include in the analysis. It is expected that relationships of depth and light on the live maerl layer would also be captured in the SST; lack of significance may therefore be due to limited statistical power of this subset of data. Only wave exposure had a significant positive relationship with the SST. This may be due to wave exposure influencing export of organic production from associated maerl fauna and flora. Wave exposure, however, would also then be expected to have a similar significant positive correlation with maerl % cover, and maerl % cover with the SST. The significant negative correlation of depth with the TST may reflect the inverse and significant positive correlations observed between depth and maerl % cover, and maerl % cover and the TST. The significant positive correlation of wave exposure and the TST, when viewed with the significant negative correlation between wave exposure and maerl % cover, suggests that the TST is largely structured by local hydrodynamic conditions rather than mediated through indirect effects on maerl habitat health. The significant positive correlation between maerl % cover and the TST shows the distinct importance of maerl habitat health on sediment carbon sequestration. The significant positive correlation of maerl % cover with the TST, and not the SST, may be the result of the greater contribution of maerl to Inorganic Carbon (IC) production than Organic Carbon (OC); with OC principally derived from associated fauna and flora. The different relationships between maerl % cover and the SST and the TST also highlights key differences of sediment composition and deposition with maerl habitat health and local abiotic conditions. This site may also experience anthropogenic fishing disturbance from trawling and dredging activity; disturbance was evident in the DDV footage. Given that trawling of the benthos would primarily affect shallow sediments, areas with higher maerl % cover at this site may be subject to more disturbance with this more frequent disturbance removing maerl from the sediment and creating a thinner shallow sediment layer^23^. When linked to variability of carbon content across sediment depths from Loss on Ignition (LOI) data^49^, this may highlight key considerations and uncertainties when maerl carbon content is generalised across different maerl habitats, both in health and location, and across sediment depth profiles^5,37^.

The Wyre maerl site is believed to be a habitat that is unique to Orkney, comprising a mixed mosaic of seagrass and maerl cover. Mixed seagrass and maerl habitats are not described under the JNCC Marine Habitat Classification^55^. Whilst this habitat subsequently provides information and data on more diverse forms of maerl habitat it is unclear how readily results may be extrapolated to wider regional/typical maerl habitats. However, PCA of the two subsets of data, Shapinsay and Wyre, suggest overlap of characteristics and potential extrapolation of Wyre maerl data to wider maerl habitats.

The boxplots, Figs. 1 and 2, highlight an almost full range of maerl % cover values in Wyre Sound, potentially only constrained by minimal SST data at 20% maerl cover. The spread of maerl habitat condition in these data suggest maerl habitat condition is structured by a gradient of abiotic factors with gradual declines across limiting environmental parameters, rather than strongly structured by one specific factor where you would more likely observe finite limits and limited variability in maerl habitat condition. Compared to Shapinsay, the Wyre subset has a more robust number of observations when adjusted for spatial autocorrelation (n = 102); it is therefore unclear if this gradient of maerl habitat health was not observed in the Shapinsay data due to data limitations, if the gradient is a result of competition with seagrass, or if abiotic factors limited maerl habitat health range. The Wyre Sound SST seems to increase then plateau after 50% cover. This could be attributable to maerl habitat and associated biodiversity reaching a maximum ecological function if these processes more strongly determine production of organic carbon, which then create a subsequent threshold of effect and organic carbon sequestration in this sediment layer. Differences between Shapinsay and Wyre site maerl sediment thickness may be the result of abiotic sediment deposition, differences between maerl and seagrass carbon sequestration composition, differences between the substrate type, or from mutualistic or competitive effects between maerl and seagrass in Wyre.

The SEMs characterise inverse relationships of abiotic factors with maerl and seagrass % cover, suggesting that the mosaic of maerl and seagrass is structured by competition influenced by abiotic factors. Abiotic factors were significant in determining both the SST and the TST. There were, however, differences between abiotic influences on the sediment layers. The SST was negatively correlated with depth and positively with wave exposure whilst the TST was positively correlated with depth and current speed. The inverse significance of depth for the different layers may again highlight key differences in sediment composition and deposition with water depth; this may be linked to allochthonous and terrigenous inputs^56^. This could also explain the change in significance of wave exposure between the SST and the TST; with deeper layers more disconnected from surface abiotic influences. The largest and most significant abiotic factor on the TST, however, was current speed. The non-significance of current speed on the SST may suggest local hydrodynamics are the key determining factors of sediment deposition, but do not influence sediment composition. Maerl % cover was found to be non-significant in determining either sediment layer. This suggests maerl % cover, and maerl associated fauna and flora, has no influence on underlying sediments. The lack of maerl % cover significance for the SST and the TST may also reflect a standard rate of maerl bed calcification that is independent of habitat health. Alternatively, it may highlight the disconnect of habitat health on these deeper stocks of sediment. Or that past a certain depth in the sediment, correlated with sequestration time, the diagenesis of refractory organic carbon reaches a limit. The wider importance of maerl in structuring the sediment, with maerl gravel comprising a substantial amount of benthic substrate in Orkney^5^, may not have been captured in these data due to a disconnect between this effect and the survey scale. Considering the wider importance of maerl and export of maerl gravel, this is a known data gap^5^ which the data presented in this study are unable to address. Potential competition with seagrass (indicated by their inverse relationships with abiotic factors) may also obscure effects of maerl habitat health on sediment thickness. Seagrass % cover had a significant relationship with the SST but not the TST. In Scotland and Orkney, seagrass habitats have been recorded to have thinner sediments of 0.5–1 m^13,57,58^. The significant correlation of seagrass % cover with the SST, a thinner sediment of between 0.5 m and 1 m, would then be more likely than a significant correlation between seagrass % cover and deeper sediment such as the TST which might be more heavily influenced by maerl % cover. This is further supported by the similarities of the SST between Shapinsay and Wyre, but a greater Shapinsay TST relative to Wyre TST; the mixed seagrass and maerl habitat of Wyre will have the TST impacted by seagrass (with generally thinner sediment than that of maerl). There may, however, also be additional benefits from associated biodiversity with seagrass % cover and increased trapping of allochthonous carbon with subsequent differences on seagrass and maerl carbon content^13^.

### Uncertainties

DDV footage was used to detail maerl and seagrass % cover. This is a relatively cost-effective and applicable small to medium scale survey method but may not be as accurate as in-situ methods using transects or quadrats. The scale of the survey, interpolation, and removal of data points with no maerl cover, are believed to be suitable mitigation for this potential uncertainty. There is a potential issue with interpolation of % cover between point locations, but there are limited options; it is unclear whether acoustic methods can determine maerl bed habitat areal extents and/or abundance^59^. Additional methods using multibeam survey data, and multispectral backscatter, could support or improve data collection for maerl % cover and reduce potential issues from interpolation^42,60^. In this study, DDV data to identify maerl % cover along survey transects was conducted at a scale that minimised potential misidentification of maerl % cover due to interpolation (see Figs. 7 and 8). Whilst there is some habitat heterogeneity, or patchiness, of maerl % cover across the maerl habitats, the general trends of maerl % cover across the landscape, and subsequent impacts on sediment thickness, would be expected to be averaged at that scale.

A large caveat of this approach is that SBP data were aligned to local core data^49^, with several options for dB and kHz settings. It is unclear whether the settings used in this study would be applicable to maerl habitats in other locations. Sediment layers (strata) from the cores were able to be identified from the SBP data, it is, however, unclear if these sediment layers (the SST and the TST) would be evident in other maerl and/or blue carbon habitats. Without core ground-truthing of SBP data, this may lead to erroneous identification of sediment strata, sediment thickness, and extrapolation of potential carbon stocks and sequestration potential. Additionally, the mixed maerl and seagrass habitat of Wyre may be unique to Orkney, though PCA of Shapinsay and Wyre data suggests they have similar properties. Cores are most likely required to support identification and corroboration of sediment layers through SBP data. Given the relative novelty of using SBP data to detail blue carbon sediment thickness, and inability of SBP data to determine sediment carbon content and source, it is unclear how readily accepted these types of data may be integrated into blue carbon accounting processes^7^.

In analogous research, seagrass TST determined from SBP data was found to be unreliable, with additional potential error due to capture of surface biomass in sediment layer extents^13^. Maerl surface biomass, however, is substantially different and less evident than seagrass surface biomass. Maerl surface biomass as the uppermost layer of 0.2–0.35 m sediment was variably evident but this was substantially thinner than seagrass surface biomass. Maerl live biomass is also included in the sediment, rather than on top of it; maerl surface biomass was therefore unlikely to have affected data analysis.

Abiotic data were available at different resolutions, with depth recorded from the SBP data, at a much finer resolution to current speed and wave exposure. It is unclear how much the differences in resolution may have affected subsequent analysis. Current speed and wave exposure data were taken from the best available source specific to Orkney^61,62^. Therefore, further mitigation of current speed and wave exposure resolution is only achieved through collection of data on a larger scale which was unfortunately outside the financial and logistical scope of this research. Residual variance in sediment thickness observed in the SEM analysis suggests there are key deterministic factors that were outside of the scope of this research; this may include other abiotic data such as geological effects^51^ terrigenous/allochthonous inputs^22,56^, and other measures of biodiversity^17,18^. The integrated approach of core, SBP, DDV, and abiotic data highlights, however, potential pathways for future research to address this key data gap.

### Future research

Future research of maerl as a blue carbon habitat is informed by the wider context of policy frameworks, logistical and financial constraints, and key data gaps. The wider context of policy frameworks must consider the carbon accreditation processes and the overarching aim of blue carbon in policy^7^. Logistical and financial constraints direct blue carbon science to the most cost-effective and robust methods of blue carbon quantification^12^ that then matches the requirements of the policy frameworks. Finally, key data gaps identified allow future research to then address areas of uncertainty, considering best methods of robust cost-effective quantification, and final implementation into policy.

SBP data will most likely only be accreditable when matched to location specific core data, and layer specific carbon content. However, with future research creating a wider knowledge base of SBP and core data, which are habitat and location specific, there would be expected to be increased confidence in this approach. This might allow SBP data, with habitat health assessments, to better define the bounds, variability, and carbon content of maerl bed sediments; this would lead to more robust accreditation and potentially higher value of those credits^7^. A key area for future research could be to identify and isolate the most suitable frequencies, or range of frequencies, with SBP to accurately measure maerl bed sediment thickness; this would increase robustness of SBP data in blue carbon research, improve confidence in the method, and alleviate financial and logistical constraints of blue carbon research. Another key area of future research could aim to quantify the links of associated maerl biodiversity and habitat health in determining organic carbon inputs into underlying sediments. Further research into integrated core, SBP, and habitat health data would then also be expected to lead into more robust machine learning applications and predictive modelling. Modelling approaches include abiotic data^5,63^, biotic data^13,45^, and might allow for greater confidence in large scale assessments^7^.

**Figure 5:**
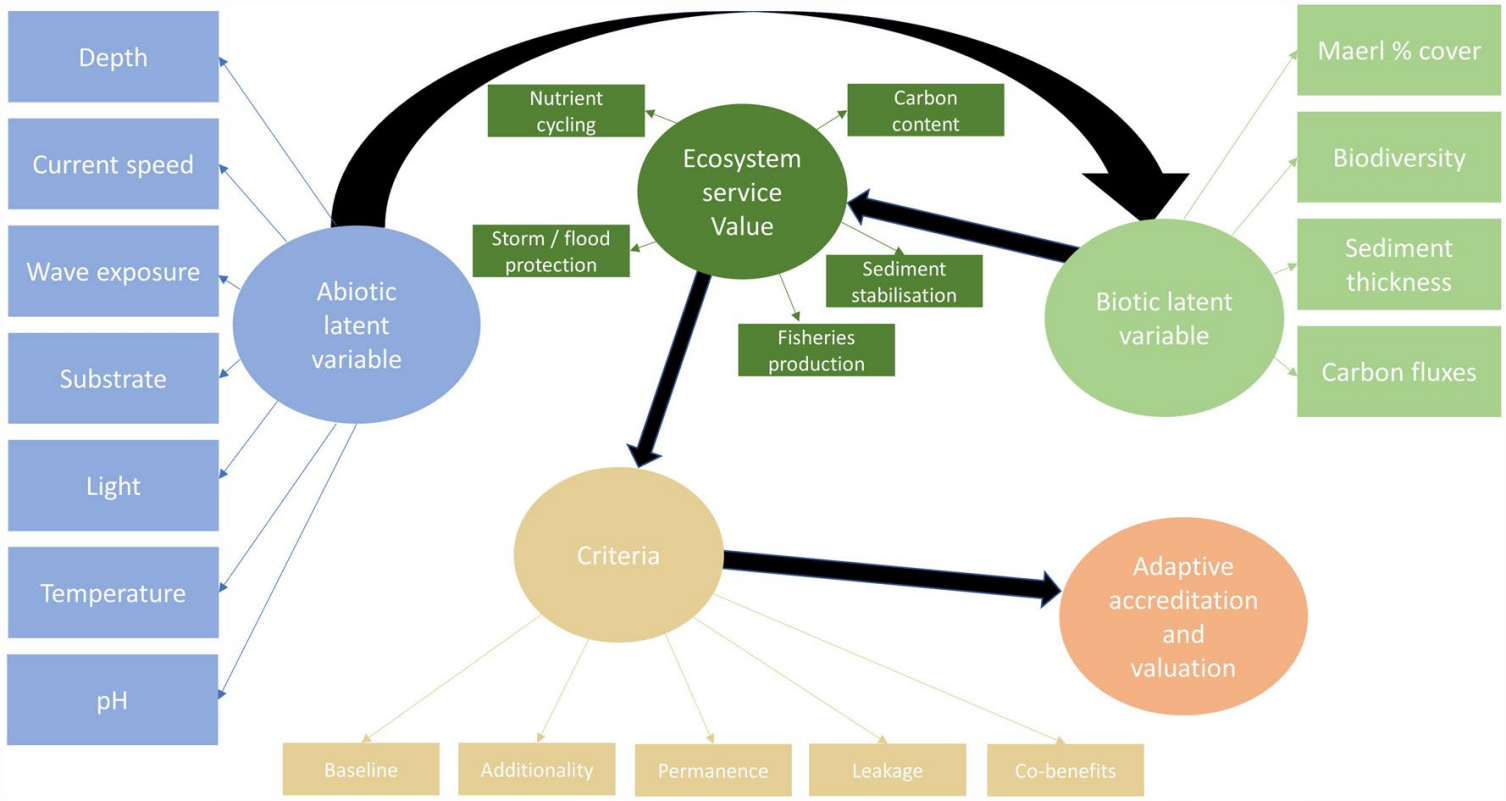
Maerl SEM for machine learning techniques to link abiotic and biotic factors to carbon accreditation criteria, adaptive valuation, and total economic value.

This feeds into considerations of financial and logistical constraints in blue carbon science. Future blue carbon research that then focuses on SBP data as a viable method of quantification further reinforces the robustness of this approach. Within a framework that uses SEM, future models may incorporate wider abiotic and biotic data to further refine understanding of the links between abiotic factors, maerl habitat health, and sediment thickness and composition. Considering ecosystem service value and accreditation criteria, latent SEM may use a similar structure to that detailed in Fig. 5.

This links a wider range of abiotic data that might be suitable at large scale, to a multitude of biotic data, ecosystem service value, accreditation criteria, and carbon credit value based on adaptive valuation^7^. Alternatively, latent variables could be structured to determine whether the maerl habitat acts as a carbon source or sink^11,21^, with temperature^16^ and pH^64^ known to be key factors.

## Conclusions

Primarily, this study evaluates the use of SBP data to identify and determine maerl bed sediment thickness. By aligning SBP data with local core data, this study details how maerl bed sediment layers can be robustly identified with SBP data. As such, this study provides a guide on how to analyse SBP data for estimation of maerl bed sediment thickness. In doing so, this study also addresses substantial data gaps on maerl bed sediment thickness, adjusting previous estimates of 0.25–0.86 m^5,22,37^ to 1.08 m for typical maerl habitats. This study therefore has the potential to increase previous maerl carbon stock quantification estimates by as much as 4 times. Considering accreditation criteria, it is unclear whether sediments with a thickness greater than 1 m may be considered as ‘additionally’ protected if disturbance is limited to the top 1 m of sediment soil^7^. The increased sediment thickness detailed in this study, however, supports the consideration of maerl beds as significant stores of marine carbon. These large stocks of carbon within maerl habitats highlights their potential carbon value, and subsequently promotes the inclusion of maerl in blue carbon frameworks to support climate change mitigation^7,8^.

This study also furthers blue carbon research that links ecological health to ecosystem function and carbon quantification^11,26,27^. This study provides the first data of its kind to highlight these links in maerl habitats, and details how maerl % cover is highly significantly correlated with TST in typical maerl habitats. In doing so, this study details a pathway for future blue carbon science based on an integrated approach of core, SBP, DDV, and abiotic data to support small to medium scale assessment of maerl habitats and carbon content^12^. This approach also suits future research needs, and provides data that are suitable for large-scale assessment based on machine learning and predictive modelling^5,63^. Subsequently, this study considers the financial and logistical constraints of future research agendas^34^, ecological values of blue carbon habitats beyond carbon sequestration^17,18^, and supports blue carbon science within the wider policy context of eco-social economics^7^.

## Methods

### Study area

**Figure 6:**
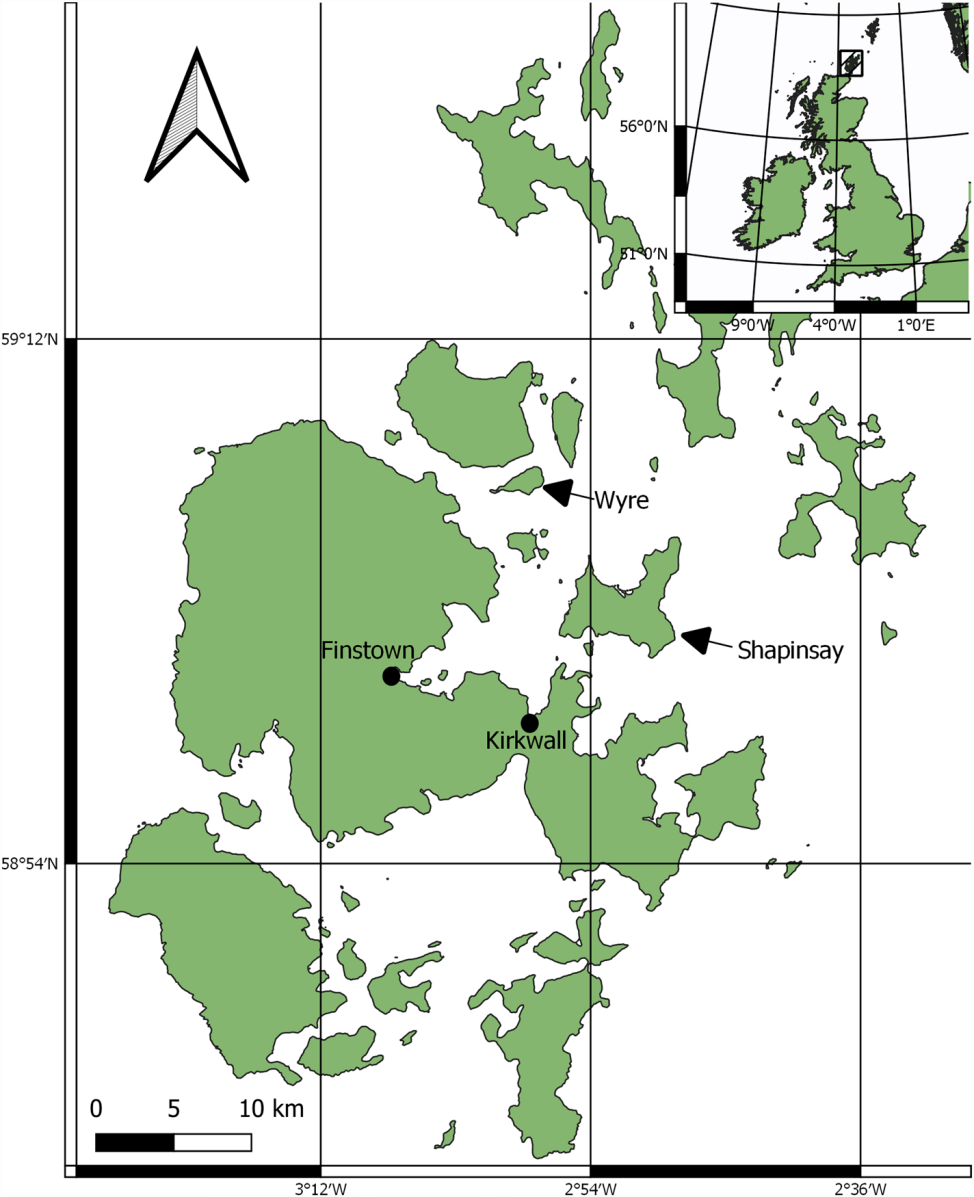
Survey area.

The survey area is focused on Orkney, Scotland as a key area of maerl habitat^5^. Key sites were identified off the eastern coast of the mainland of Orkney near the isles of Wyre and Shapinsay. The survey area is defined in Fig. 6.

Suitable locations to survey maerl habitats were identified from Maximum Entropy (MAXENT) predicted models^5^, online records of maerl habitats^65^, and local expertise and knowledge; survey locations were also aligned with existing core data^49^. Key locations were identified south of Shapinsay (59° 01′ 22.1″ N 2° 51′ 06.1″ W), in Wyre Sound, just north of Wyre (59° 07′ 29.0″ N 2° 59′ 23.2″ W), and to the south of Wyre (59° 06′ 43.6″ N 2° 58′ 51.7″ W). The Shapinsay site features a mixed sediment substrate^52^ with only maerl presence and is hereafter referred to as the Shapinsay Maerl (SHM) site. The Wyre survey sites, however, feature maerl that exists alongside seagrass on a coarse substrate made up partially of dead maerl gravel; seagrass and maerl habitats overlap with seagrass interspersed in the maerl habitat, snapshots taken from DDV data to show this are available in the Supplementary material. There are three distinct areas to the Wyre survey sites. The first was a relatively evenly mixed seagrass and maerl (WSGM) site south of Wyre, the second was in Wyre Sound on a predominantly maerl habitat with patchy seagrass (WM), and the third site (WF) was at the east end of Wyre Sound which also featured a predominantly maerl habitat with limited seagrass presence. The third site (WF) was designated WF for Wyre flame shell, *Limaria hians*, as National Biodiversity Network (NBN) records^66^ indicated potential flame shell presence here; flame shell presence at the WF site, however, was unable to be ascertained from DDV data. All sites feature a mosaic of maerl abundance with associated fauna and flora.

Maerl beds around Shapinsay were in ~ 15 m of water, whilst maerl beds around Wyre were in much shallower depths of ~ 6–10 m in Wyre sound and ~ 4–6 m depths south of Wyre. The tidal range for Kirkwall, the nearest port, is 3.2 m; Kirkwall has a maximum tidal height of 3.4 m and minimum of − 0.2 m^67^.

### Survey design and protocol

This study collated regional abiotic data^61,62^ and local core data^49,51^ to support the research aims. The survey collected SBP data with which to detail seabed depth (at fine scale) and sediment thickness, in addition to drop-down video (DDV) data to detail maerl and seagrass % cover. Survey data collection was conducted on MV Challenger with the help of a local marine operations consultancy team with previous experience in SBP operation and data collection^68^.

Maerl presence at each site was first confirmed with DDV footage. Once confirmed, transect survey lines were set as the vessel drifted with local water currents; this maintained vessel speeds of < 4 knts and avoided potential propellor induced disturbance on SBP data. Transect survey lines were set in conjunction with tide times to allow replication of survey lines and for tide adjustment of depth data in subsequent analysis. Maerl and seagrass % cover was recorded in real time from review of the DDV data at discrete points along the transect survey lines. Global Positioning Systems (GPS) data from the boat track plotter were used to align DDV footage with location. DDV data and maerl and seagrass % cover values were additionally checked in desktop data review.

Three suitable survey transect lines were determined for the Shapinsay maerl (SHM) survey site; SHM1, SHM2, and SHM3. Suitable survey lines were also set for each of the Wyre survey sites; WSGM1, WM1, and WF1. Core data were aligned to all survey sites; cores WM 1A, 1B, and 1C^49^ were aligned with the WM site, and cores WF 2A, 2B, and 2C^49^ with the WF site.

SBP surveys were then conducted on the transect lines using the same method of current drift with the DDV. GPS positioning data were recorded directly from the SBP. Data were recorded with an Innomar Compact SES 2000^69^, fixed to the side of the boat using a bespoke attachment pole for SBP surveys. Due to the novel nature of this approach for blue carbon sediment thickness estimation, multiple operating frequencies were used. Transect lines were surveyed twice, simultaneously at Low Frequency (LF) at 8 kHz at − 6 dB and high frequency (HF) at 10 kHz and 7 dB, and then again using the multi-frequency (MF) setting which simultaneously recorded data LF data (at − 6 dB) and HF data (at 7 dB) at 5 kHz, 10 kHz, and 15 kHz. All data were recorded in RAW file format to capture all potential data from the survey. All SBP data channels and settings are detailed in the Supplementary material.

**Figure 7:**
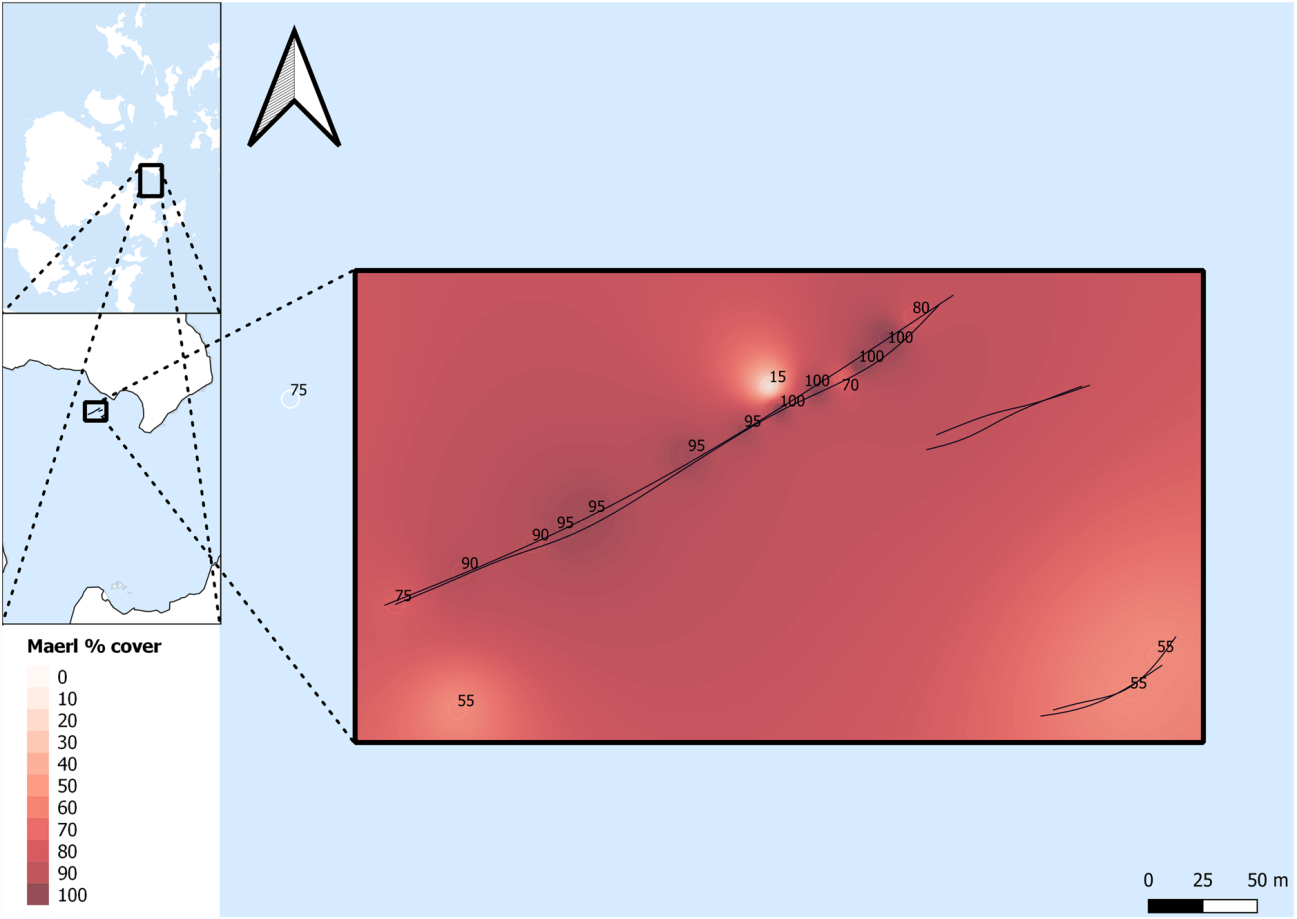
Shapinsay survey transects. SHM11 and SHM12 (top), SHM21 and SHM22 (middle), and SHM31 and SHM32 (bottom). Waypoint maerl % cover is noted in black text along the transects.

**Figure 8:**
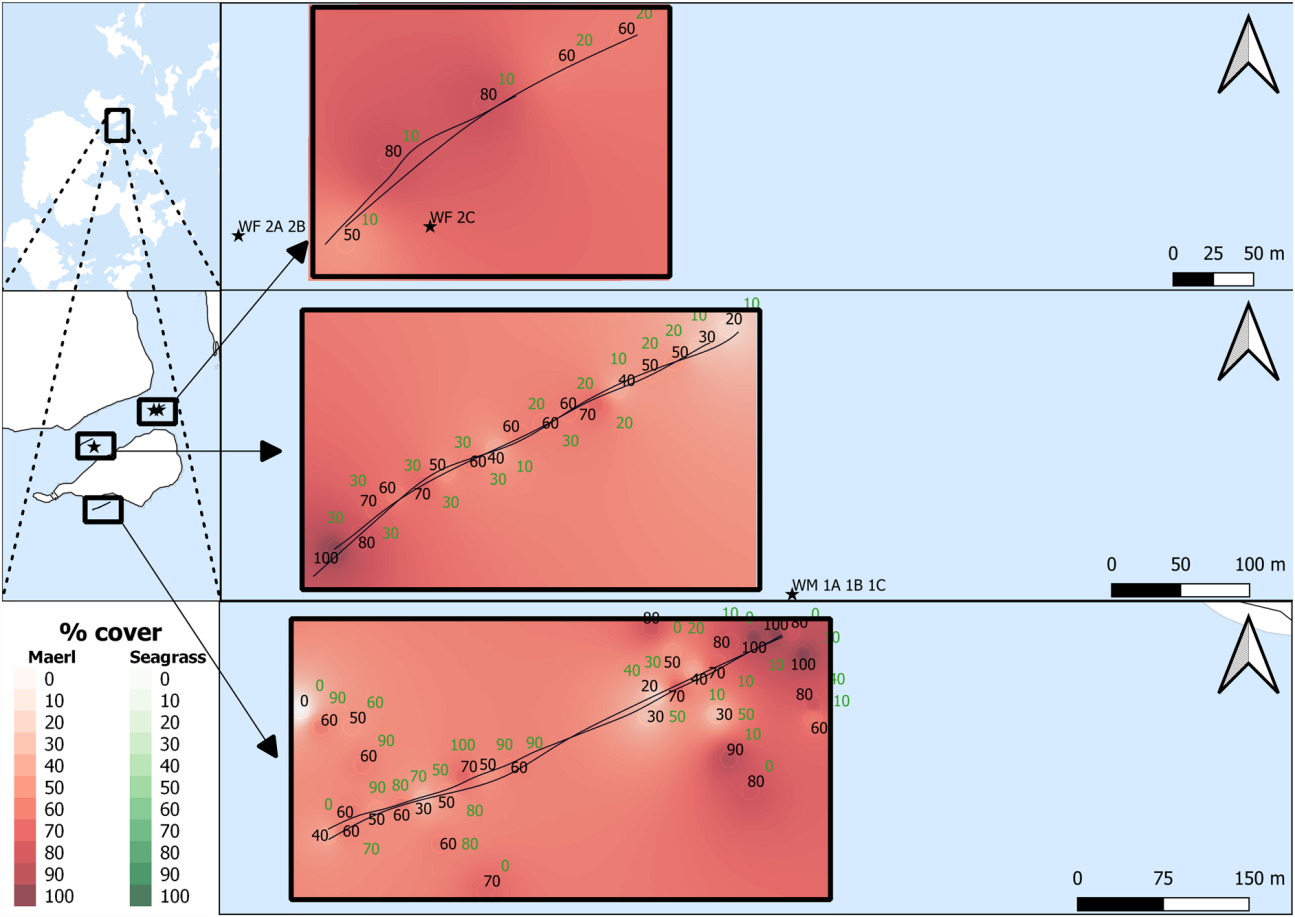
Wyre survey transects. WF11 and WF12 top, WM11 and WM12 (middle), and WSGM11 and WSGM12 (bottom). Survey core data for WF 2A, 2B, and 2C (top) and WM 1A, 1B, and 1C (middle). Waypoint maerl % cover is noted in black text, with seagrass % cover noted in green text, along the transects.

Survey data were named per transect line, i.e., SHM11 and SHM12 for the two SBP transects, one with LF and HF, and one using MF, conducted on SHM1. All SBP transects are listed as SHM11, SHM12, SHM21, SHM22, SHM31, SHM32, WM11, WM12, WF11, WF12, WSGM11, and WSGM12. Survey transects and survey areas are detailed in Figs. 7 and 8

### Data alignment with % cover

Maerl and seagrass % cover, estimated visually from DDV data, were imported with GPS positions into QGIS^70^. DDV data were collected across the whole transect, with observable changes in % cover recorded with the location marked as a waypoint; waypoint % cover data are detailed in Figs. 7 and 8. Maerl and seagrass % cover values were interpolated using the inverse distance weighted (IDW) method to convert point data to contiguous map data. Data points with no maerl cover were removed in the SEM analysis to avoid potential bias of data due to inaccurate identification of maerl habitats. At ranges of low maerl % cover, identification of maerl % cover was particularly difficult, with additional complications from trying to assess live and dead maerl on a dead maerl substrate from DDV data. Whilst a comparative identification of no maerl cover sediment against maerl habitat sediment thickness would have been interesting, the difficulty of accurately identifying no maerl and low maerl % cover suggested a more robust approach was to remove these data from subsequent analysis. SBP data were converted to SEGY file format using Innomar SES Convert software^71^. SEGY data .txt files, with GPS ping data, were also imported into QGIS then aligned to interpolated maerl and seagrass % cover values. Data were exported from QGIS as .txt files to allow import into SeiSee software^72^. This allowed for % cover values to be added to SEGY data and analysed with SBP data in Sonarwiz seafloor mapping and analysis software^73^.

### Alignment with core data

Core data, from maerl habitats in Wyre Sound, were reviewed from analogous research^49^ alongside other regional core data^51,57,58^, and wider research on acoustic data for blue carbon sediment quantification^38–40^. Patterns of sediment stratification from Wyre Sound core data^49^ were identified from Loss on Ignition (LOI) data, particle size analysis, and visual inspection. Cores contained live maerl from the sediment surface to 0.35 m depth; WM cores 1A, 1B, and 1C exhibited live maerl up to a sediment depth of 0.2 m, whilst WF cores 2A, 2B, and 2C contained live maerl up to a sediment depth of 0.35 m. All cores had a layer of shell fragments at sediment depths of 0.7–0.8 m. Both Organic Matter (OM) and Inorganic Carbon (IC) were correlated with depth, OM was generally negatively correlated with depth whilst IC had an inverse positive correlation with depth, with an additional distinct composition of OM and IC separated around the shell fragment layer at 0.7–0.8 m sediment depth; core data had a maximum thickness of 1.3 m at the WM site^49^. Similar layers in blue carbon sediments are reported in wider research^13,45,63^.

SBP data and aligned core data were reviewed in Sonarwiz with exposure, gain, and colour settings set to highlight sediment layers identified in the core data. Bottom tracking, using the ‘threshold detection’ algorithm, was used to detail the seabed in SBP files; blanking, duration, and threshold settings were adjusted per file for best fit. In addition to this seabed reflector, first multiples and bedrock were also identified and highlighted where applicable to further help with sediment layer identification. First multiples are reflections of the SBP pings hitting the seafloor repeated at twice the travel time to reach the seafloor; these were identified by selecting m/s travel time annotation in the preferences of image analysis in Sonarwiz. Sediment layers, identified and matched to the core data, were added as acoustic layers within the maerl survey transect files. Layers were identified with varying resolution and definition dependent on the SBP setting, with different results observed due to differences in channel dB and kHz ranges. Layer sediment thickness with reference to the seabed was determined using the ‘calculate thickness’ feature in Sonarwiz. Data were then exported as a .csv file, with associated maerl % cover, seagrass % cover, and depth and imported back into QGIS. Best available Orkney specific current speed^61^, and wave exposure^62^ data were matched to SBP ping data by GPS position in QGIS then exported for subsequent analysis in R^74^.

**Figure 9:**
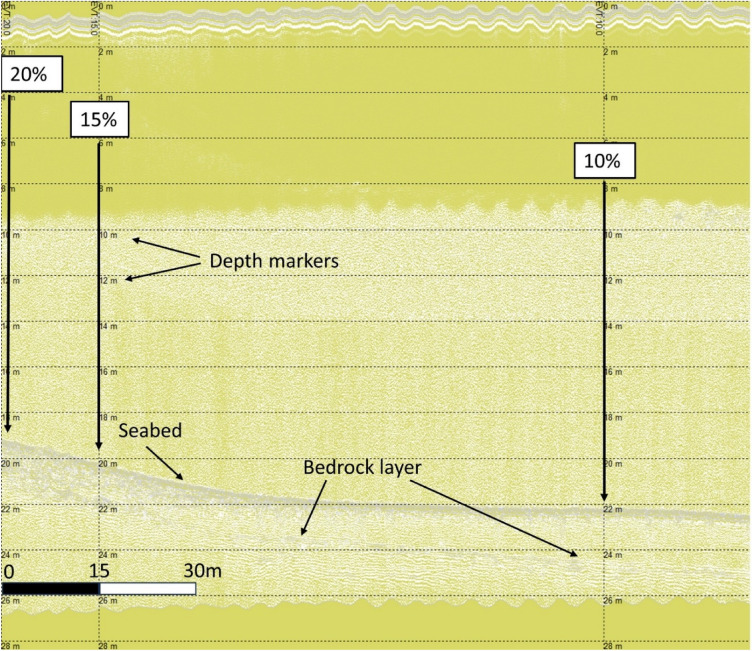
Shapinsay horse mussel LF data with clear identification of underlying bedrock.

The settings used on analysis of SBP data in Sonarwiz exhibited variable results for sediment layer identification. Two potential layers, up to shell fragment layer and after shell fragments to total sediment thickness^49^, were most evident on two specific SBP channels. The shallow layer, up to shell fragments around 0.7–0.8 m and hereafter referred to as the Shallow Sediment Thickness (SST), was most readily evident on transects using the HF1 channel (7 db at 5 kHz). The shallow live maerl layer from the sediment surface down to 0.2–0.35 m was also partially evident at the top of the sediment on this channel, however, given the fine scale nature of this layer, and SBP data noise, identification of this layer was not sufficiently robust for further analysis. The deeper layer beyond the shell fragments at 0.7–0.8 m depth in the sediment, aligned to total sediment thickness and hereafter referred to as the Total Sediment Thickness (TST), was best aligned to the LF3 channel (− 6 dB at 15 kHz). First multiplies were generally evident on all channels, whilst the LF channel (− 6 dB at 8 kHz) was found to be the most viable for identifying bedrock underlying sediment layers. An example of data alignment of % cover, depth, seabed, and bedrock identification, from an analogous study on horse mussels^45^, is shown in Fig. 9.

**Figure 10:**
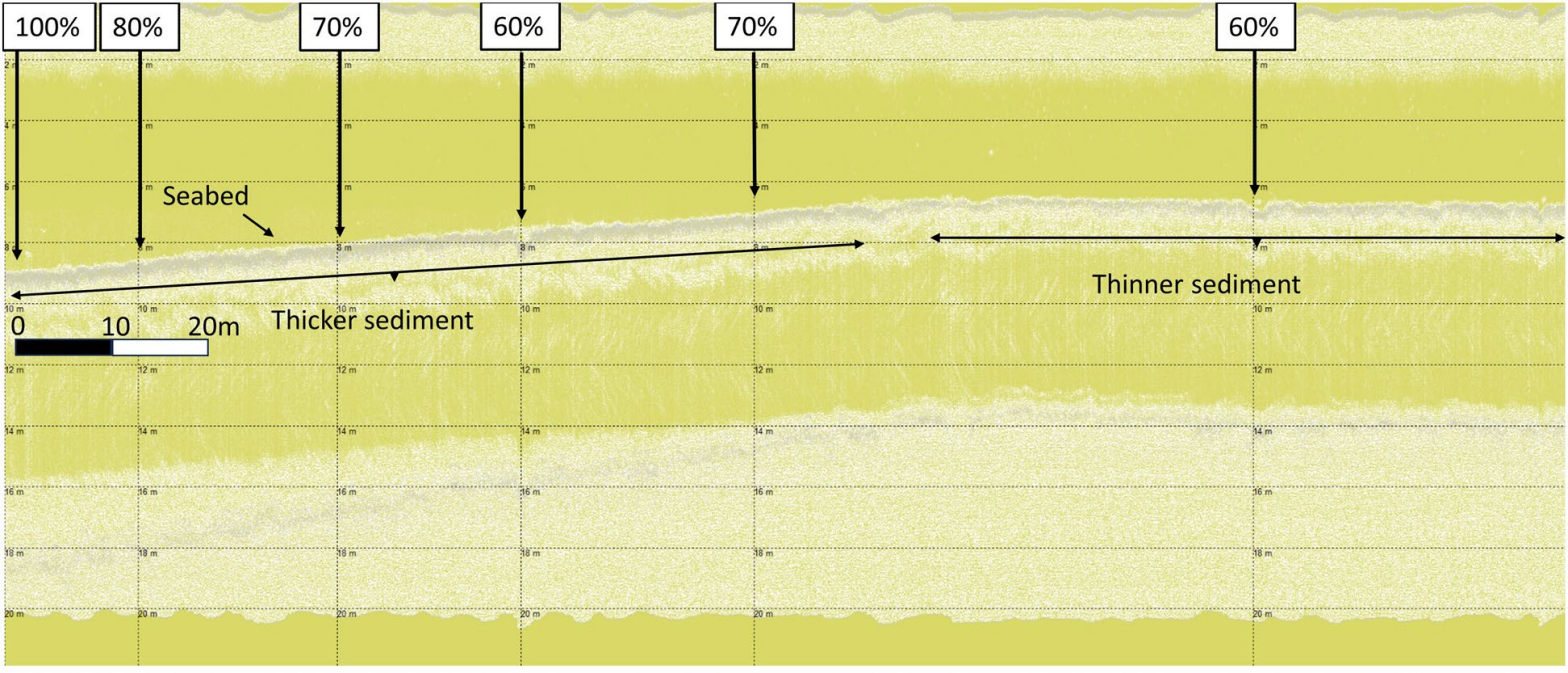
Wyre maerl (WM12) HF data with sediment thickness variability.

Identification of varying sediment thickness on HF channels (7 dB at 10 kHz) further supported conclusions that layers identified were not erroneous interpretations of signal noise from the SBP pulse signature, but instead corroborated specific sediments. Examples of layer thickness variability, rather than monotone SBP noise, and linked to maerl % cover, can be seen in Fig. 10. From left to right in Fig. 10, maerl % cover changes from 100% down to 60%, briefly fluctuating back up to 70% from 60% before decreasing down to 60% again. On the left side of the image, in conjunction with greater % maerl cover, thicker sediment is evident in relation to that to the right side of the image, aligned with 60–70% maerl cover.

**Figure 11:**
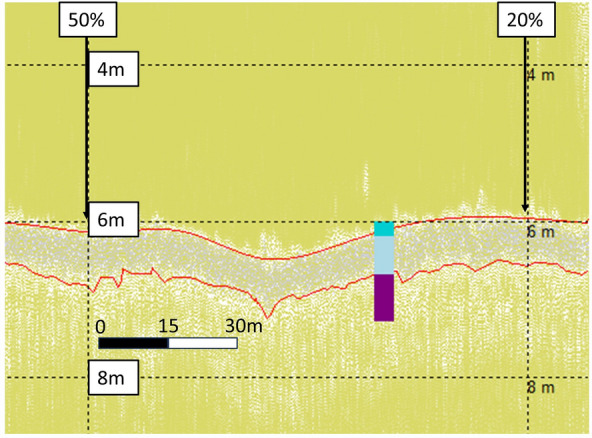
Wyre Maerl (WM11) HF1 data with aligned core data (cores WM 1A, 1B, and 1C) up to shell fragments, and termed the Shallow Sediment Thickness (SST), at 0.7 m^49^. Top 0.2 m layer of live maerl in dark turquoise, mixed maerl and gravel layer up to SST at 0.7 m in light blue, and anoxic fine mud and gravel layer down to Total Sediment Thickness (TST) of 1.3 m in purple. Top red line highlights the seabed, bottom red line highlights the SST identified in the core.

**Figure 12:**
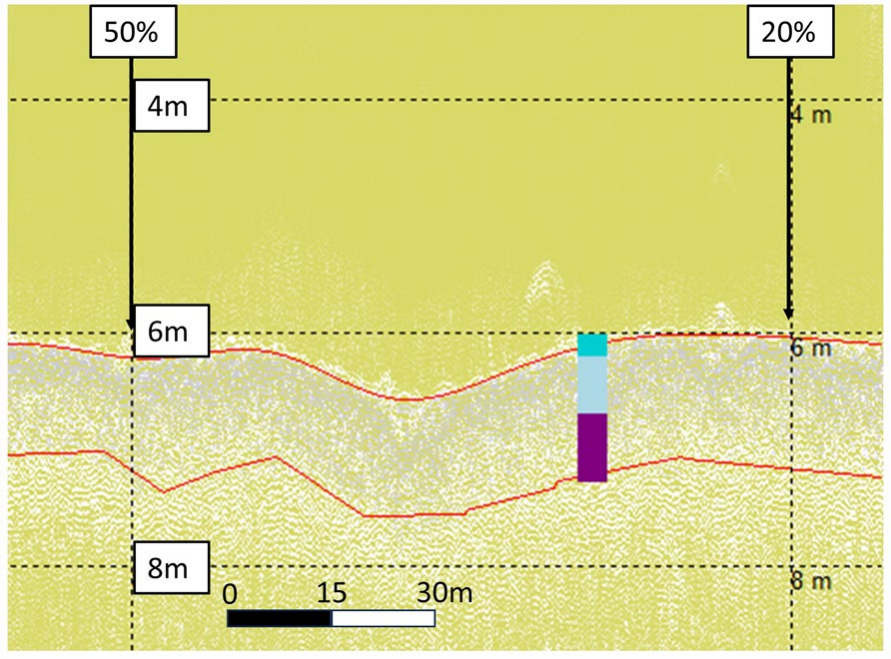
Wyre Maerl (WM11) LF3 data with aligned core data (cores WM 1A, 1B, and 1C) up to Total Sediment Thickness (TST) at 1.3 m^49^. Top 0.2 m layer of live maerl in dark turquoise, mixed maerl and gravel layer up to Shallow Sediment Thickness (SST) at 0.7 m in light blue, and anoxic fine mud and gravel layer down to TST of 1.3 m in purple. Top red line highlights the seabed, bottom red line highlights the TST identified from the core.

Maerl sediments are characterised by bands of light and dark grey on the SBP data. Particularly on the HF1 channel, a light grey band at the top of the sediment/seabed aligned to records of live maerl depths^49^ but could not be reliably identified. Identifying the dark grey band, however, was more feasible and definite. This dark grey band was most closely aligned to the SST at 0.7 m on the HF1 channel, and to the TST from 1.0 to 1.3 m, on the LF3 channel^49^. HF1 and LF3 channel data showing the live maerl, SST up to shell fragments at ~ 0.7 m sediment depth, and the TST at a sediment depth of ~ 1.3 m, can be seen in WM sites in Figs. 11 and 12 respectively.

**Figure 13:**
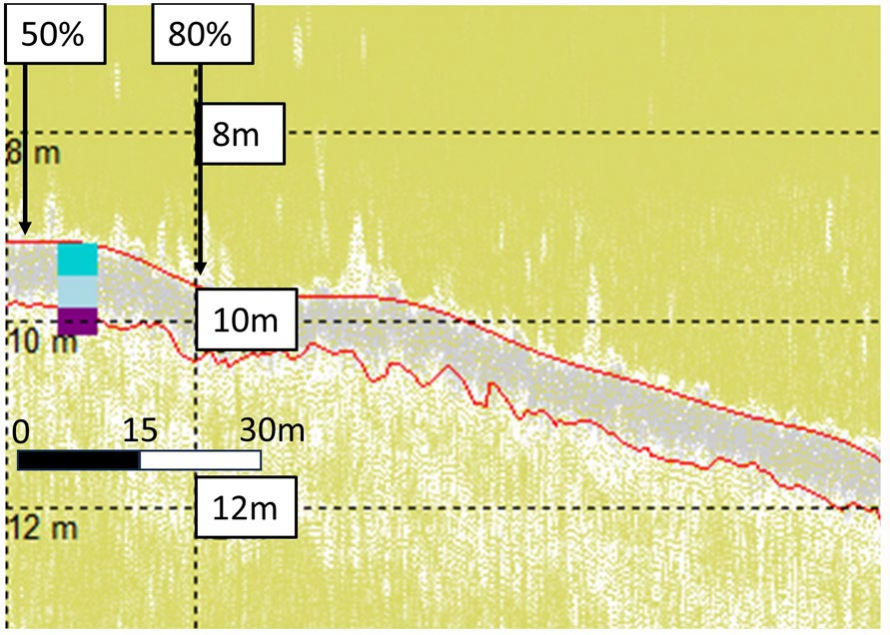
WF12 HF1 data with aligned core data (cores WF 2A, 2B, and 2C) up to shell fragments, and termed the Shallow Sediment Thickness (SST), at 0.7 m^49^. Top 0.35 m layer of live maerl in dark turquoise, mixed maerl and gravel layer up to SST at 0.7 m in light blue, and anoxic fine mud and gravel layer down to Total Sediment Thickness (TST) of 1.0 m in purple. Top red line highlights the seabed, bottom red line highlights the SST identified in the core.

**Figure 14:**
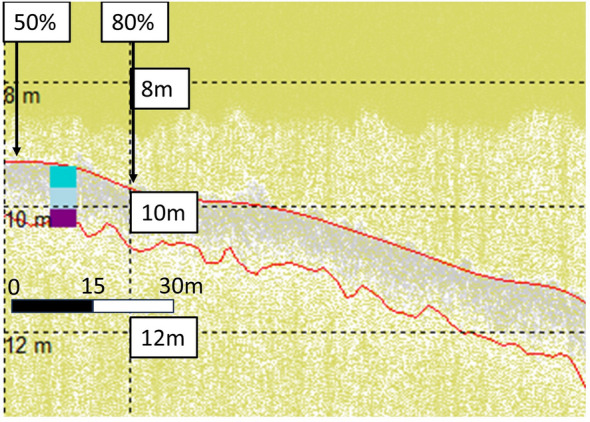
WF12 LF3 data with aligned core data (cores WF 2A, 2B, and 2C) up to Total Sediment Thickness (TST) at 1.0 m^49^. Top 0.35 m layer of live maerl in dark turquoise, mixed maerl and gravel layer up to SST at 0.7 m in light blue, and anoxic fine mud and gravel layer down to TST of 1.0 m in purple. Top red line highlights the seabed, bottom red line highlights the TST identified from the core.

The alignment of the SST on the HF1 channel, up to shell fragments at 0.7 m sediment depth, and TST of 1.0 m on the LF3 channel can be seen in WF survey data in Fig. 13 and Fig. 14 respectively.

Variables were tested for covariance and to determine best data; depth and current data were available from two different sources. Covariance and scaled covariance of principal components are provided in the Supplementary material. Principal Component Analysis (PCA) of the two subsets of data was also used to investigate differences between the two sites; Wyre and Shapinsay data. The PCA revealed site similarities and therefore subsequent suitability for extrapolation of data analysis between the sites. Given the key addition of seagrass exclusively to Wyre sites, however, subsets of data were analysed individually.

SBP data were assessed for spatial autocorrelation; average ping rate was 4 pings per second with each ping counting as a separate data point. Using the ‘points to path’ and ‘QChainage’ functions in QGIS, with data projected in EPSG:32630 to provide accurate distance, SBP transect data were subset at distance intervals of 1 m. Data at varying intervals were then assessed for spatial autocorrelation using a mix of Moran’s test and semi-variogram testing; this dual method can better pinpoint spatial autocorrelation coefficients than single method testing^75,76^. Data were generally found to exhibit non-significance in spatial autocorrelation tests at 5 m sample distance intervals in Moran tests. Two transects were better analysed in semi-variograms and sills were identified at 10 m sample distance intervals. A sampling distance interval of 10 m was set for all data for subsequent data analysis through Structural Equation Modelling (SEM). SEM was used to determine the relative influences and interactions of abiotic and biotic factors on maerl bed sediment thickness.

Final data were comprised of SBP data which contained latitude, longitude, a transect identifier, the SBP channel, sediment thickness, and depth (taken from the SBP data and adjusted for tide). DDV data comprised maerl % cover and seagrass % cover matched to each SBP data ping record. Finally, environmental data which were the most specific to Orkney were comprised of current speed^61^, wave exposure^62^, and substrate type^52,77^; substrate type was also corroborated for each transect from the DDV footage.

SEM pathway analysis was used to determine and highlight the distinct effects of abiotic and biotic variables on sediment thickness. For this purpose, SEM pathway analysis is better suited than generalised linear models (GLMs), Generalised Additive Models (GAMs), or other regression as the SEM pathway plots can clearly differentiate the coefficient loadings involved in the analysis and perform potentially independent multiple regressions simultaneously^78^. SEM pathway analysis undertaken for this study used a priori assumptions that environmental variables determine seagrass and maerl % cover, that both environmental variables and % cover determine sediment thickness, and that these are one-way relationships, for example, sediment thickness does not determine % cover. SEM pathway analysis was conducted using the R package ‘lavaan’ version 0.6–15^79^. Saturated models (df = 0) were used rather than over-identified models due to subset data limitations given the number of included parameters^80^. Maximum likelihood with robust standard errors (MLR) was used to deal with non-normality in the data subsets^81^.

### Supplementary Information

Supplementary Information.

## Data Availability

The datasets used and/or analysed during the current study available from the corresponding author on reasonable request.
