# Supplementary material for: Sounding out maerl sediment thickness: an integrated data approach
Source: Sci Rep. 2024 Mar 3;14:5220. doi: 10.1038/s41598-024-55324-x (PMC10909873; doi:10.1038/s41598-024-55324-x)
Supplement: Supplementary file 1 — Supplementary Information. [file 41598_2024_55324_MOESM1_ESM.docx]

# Supplementary material

## Abiotic data


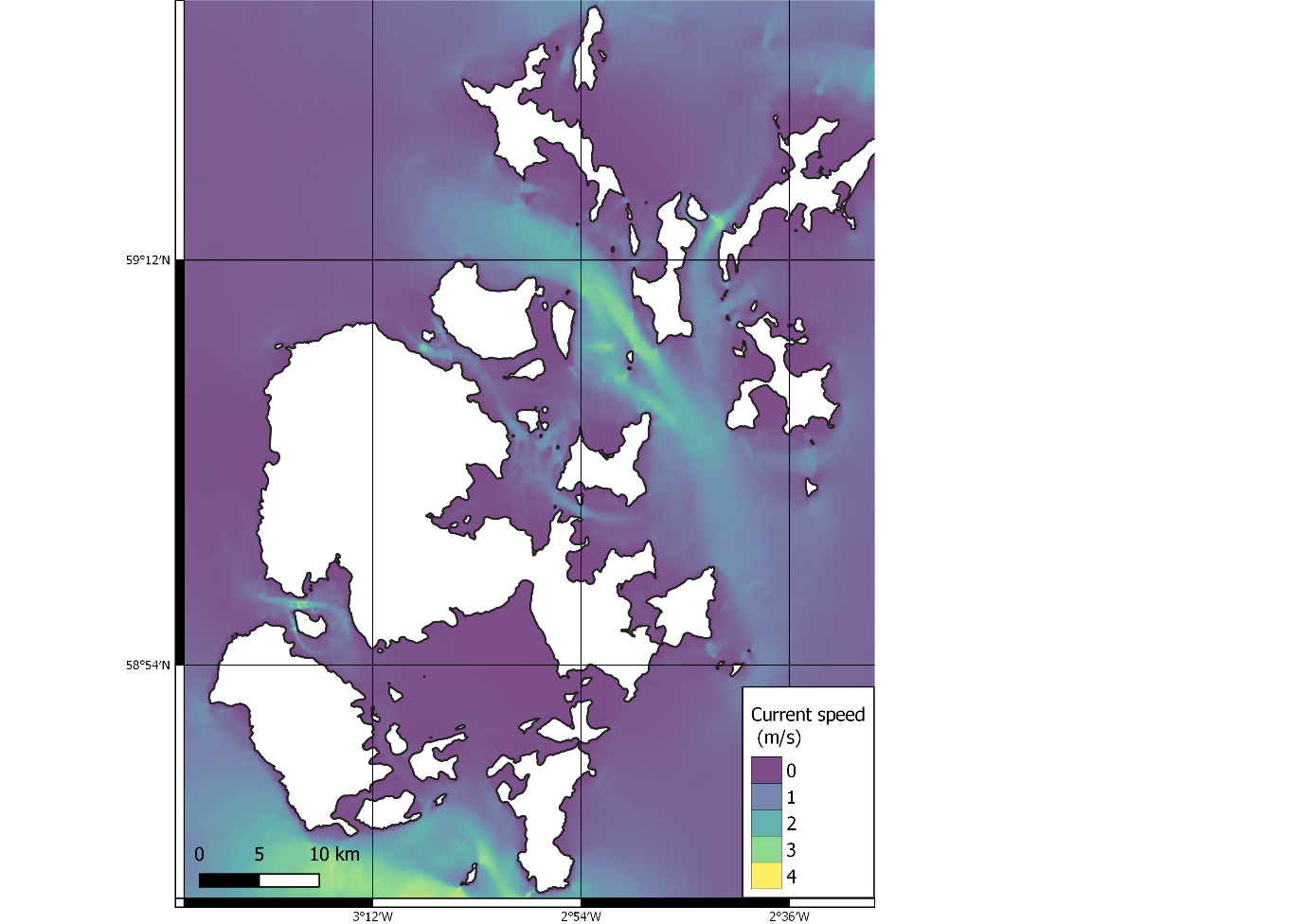


Figure 1 Orkney current speed data (Almoghayer, 2022).


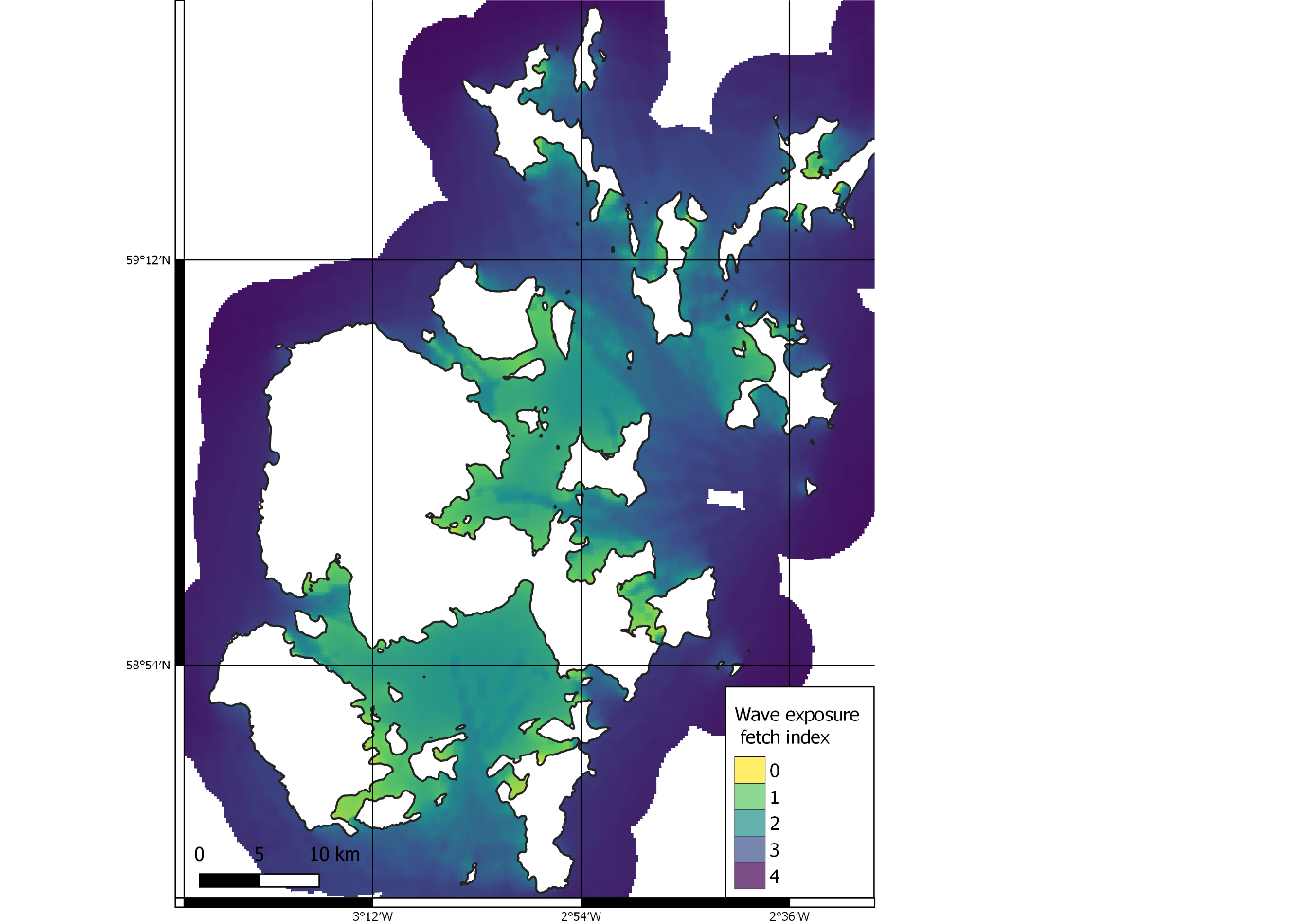


Figure 2 Orkney wave exposure data (Burrows, 2007).

Sub-Bottom Profiler (SBP) settings

Table 1 SBP data channel frequency and gain settings

| SBP channel | Sound velocity (m/s) | Frequency (kHz) | Gain (dB) |
| --- | --- | --- | --- |
| LF | 1,500 | 8 | -6 |
| LF1 | 1,500 | 5 | -6 |
| LF2 | 1,500 | 10 | -6 |
| LF3 | 1,500 | 15 | -6 |
| HF | 1,500 | 10 | 7 |
| HF1 | 1,500 | 5 | 7 |
| HF2 | 1,500 | 10 | 7 |
| HF3 | 1,500 | 15 | 7 |

## Site data


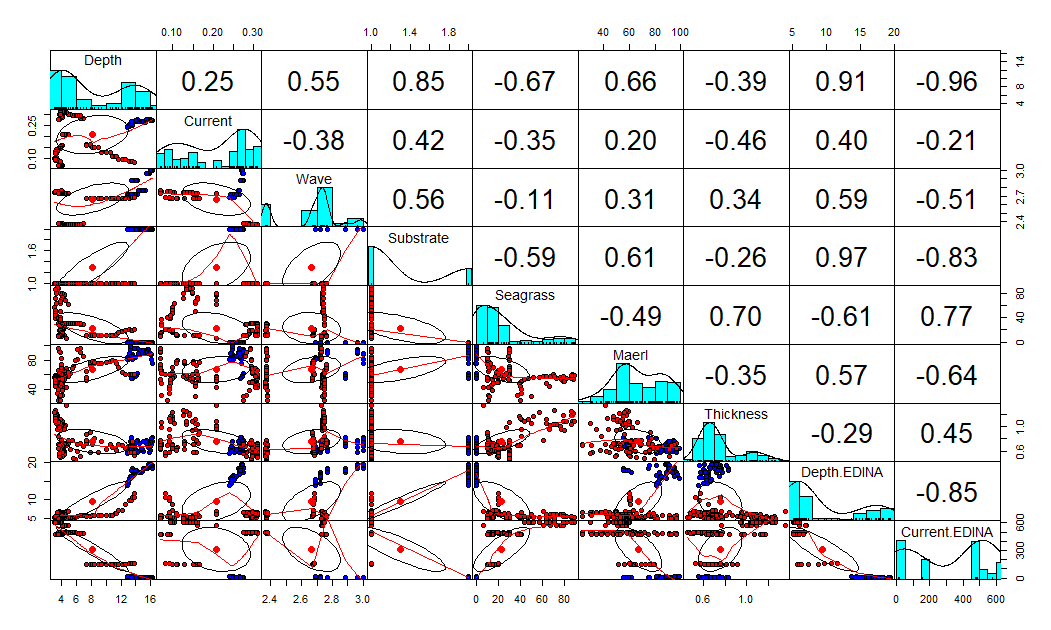


Figure 3 Survey data covariance.


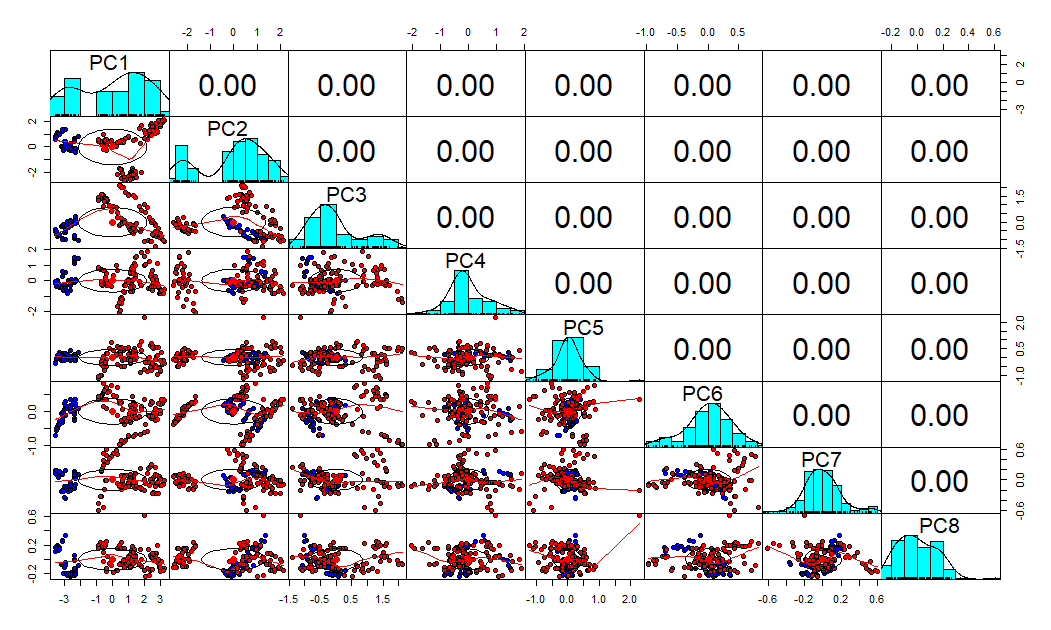


Figure 4 Scaled survey data of principal components.


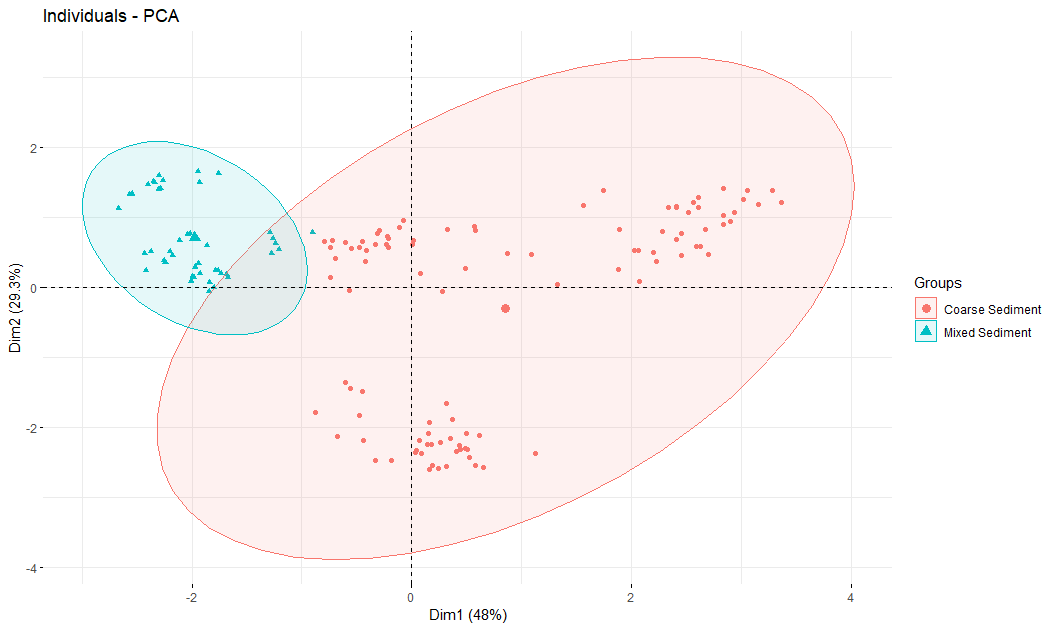


Figure 5 Principal Component Analysis of survey data based on mixed sediment (Shapinsay maerl site data) and coarse sediment (Wyre maerl and seagrass site data).

## Spatial autocorrelation tests


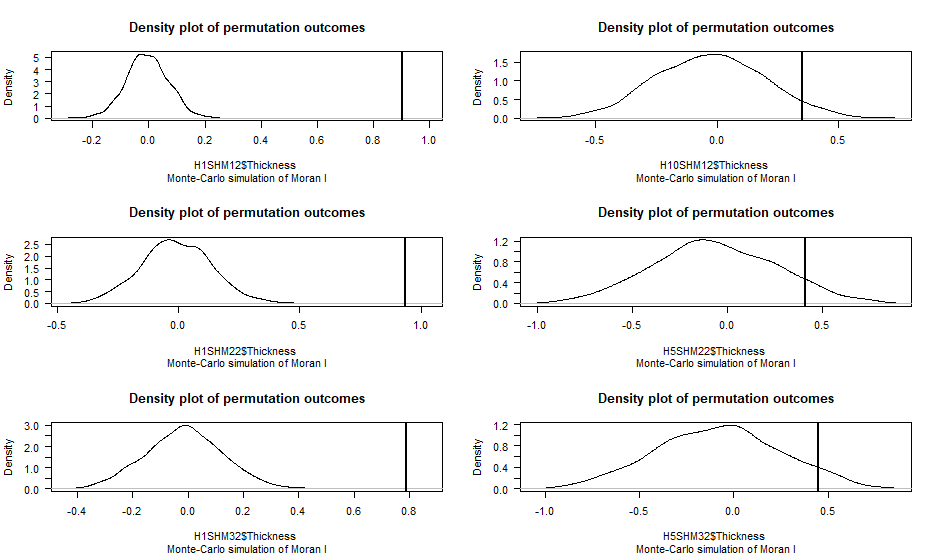


Figure 6 SHM Moran Monte-Carlo simulations at 1m (left) and 5m / 10m sampling distance intervals. SHM12 top, SHM22 middle, and SHM32 bottom.


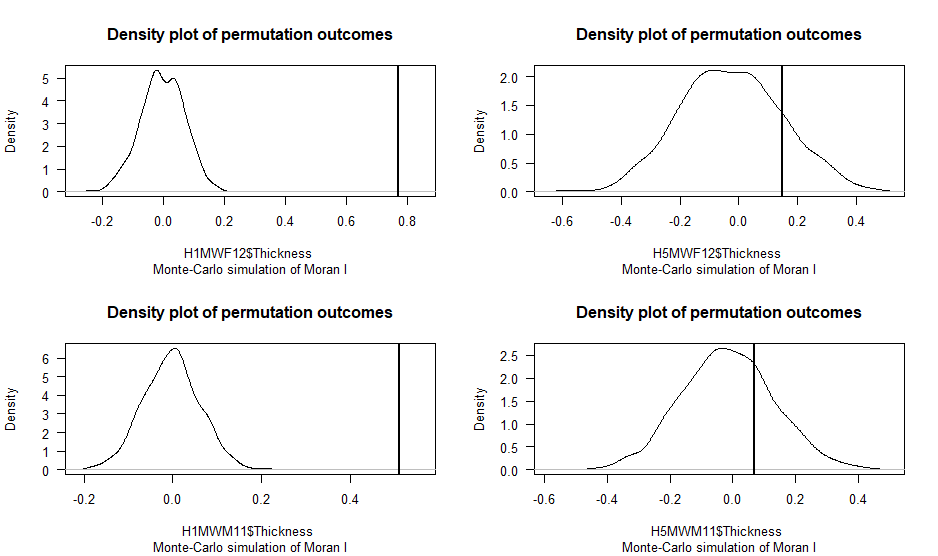


Figure 7 WF12 (top) and WM11 (bottom) Moran Monte-Carlo simulations at 1m (left) and 5m (right) sampling distance intervals.


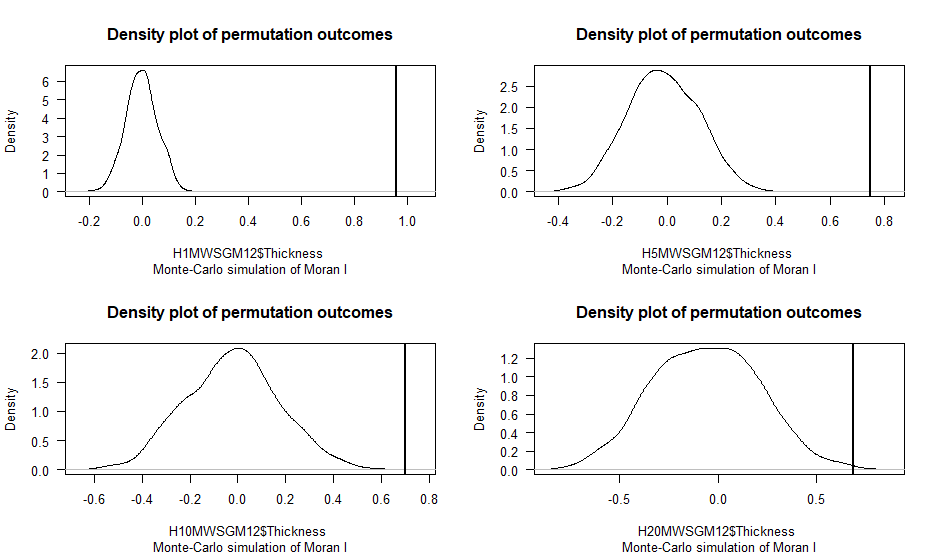


Figure 8 WSGM12 Monte-Carlo simulations at 1m (top left), 5m (top right), 10m (bottom left), and 20m (bottom right) sampling distance interval intervals.


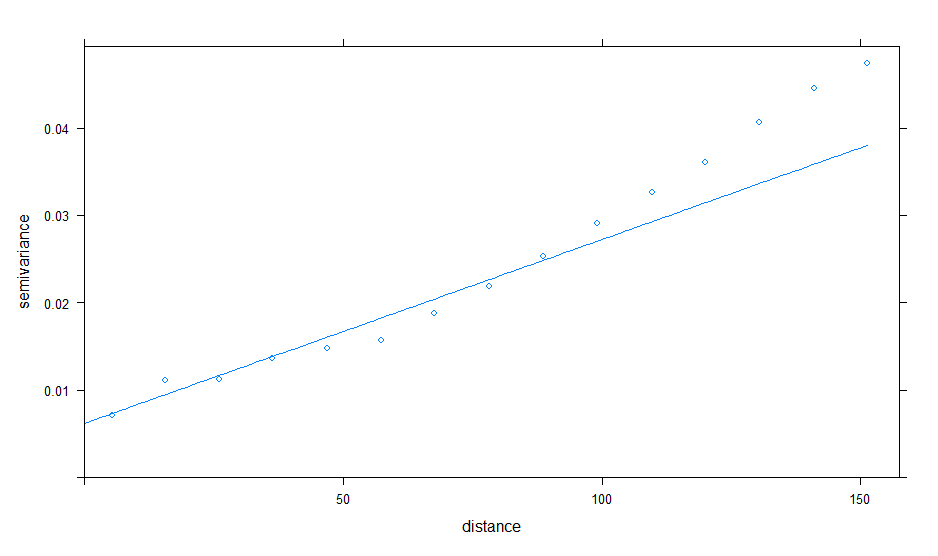


Figure 9 WSGM12 semi-variogram.

## Drop Down Video (DDV) screenshots


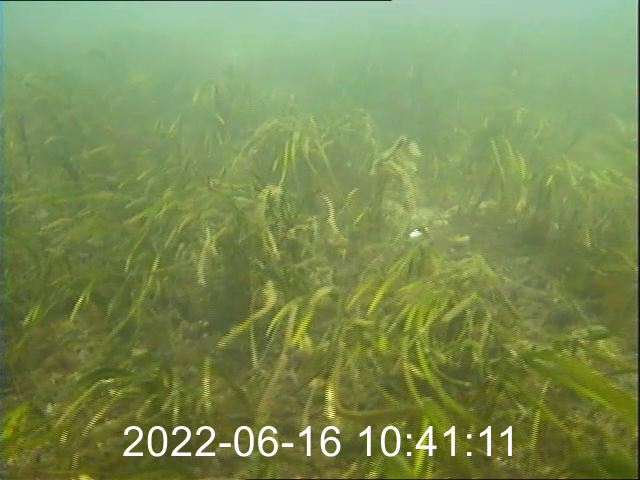


Figure 10 Wyre Seagrass and Maerl (WSGM11) DDV footage.


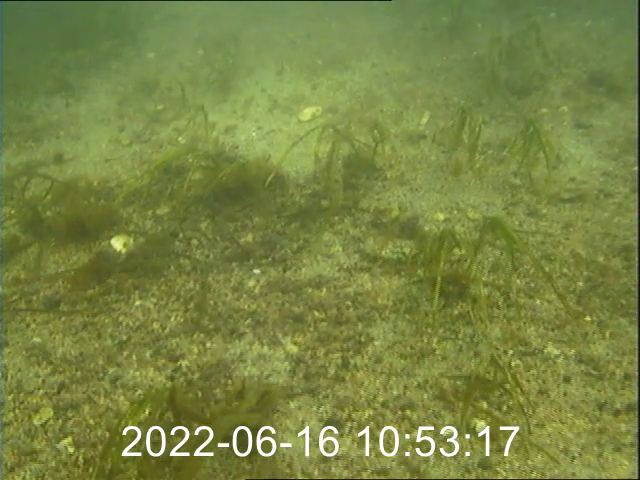


Figure 11 Wyre Seagrass and Maerl (WSGM11) DDV footage..
